# Treatment Options for Nosocomial Ventriculitis/Meningitis: A Case Report and Review of the Literature

**DOI:** 10.3390/pathogens14010003

**Published:** 2024-12-26

**Authors:** Marios Karvouniaris, Zoi Aidoni, Eleni Gkeka, Stella Niki Primikyri, Konstantinos Pagioulas, Elena Argiriadou

**Affiliations:** Intensive Care Unit, Department of Anesthesiology and Critical Care, AHEPA University Hospital, Aristotle University of Thessaloniki, 54636 Thessaloniki, Greece; zoiaidoni@yahoo.com (Z.A.); gekhel@gmail.com (E.G.); stellaprimikyri@hotmail.com (S.N.P.); argiriadou@auth.gr (E.A.)

**Keywords:** ventriculitis, meningitis, *Enterococcus faecalis*, ampicillin, ceftriaxone, nosocomial, post-neurosurgical

## Abstract

Ventriculo-meningitis or nosocomial meningitis/ventriculitis is a severe nosocomial infection that is associated with devastating neurological sequelae. The cerebrospinal fluid isolates associated with the infection can be Gram-positive or -negative, while the *Enterococcus* spp. is rarely identified. We report a case of a 68-year-old woman with a past medical history of insulin-dependent diabetes mellitus, hypertension, and coronary artery disease. She was admitted to the intensive care unit following a scheduled sphenoid wing meningioma resection. Her course was complicated with left middle cerebral artery pseudoaneurysm and hemispheric hemorrhage, and an arterial stent and external ventricular drainage catheter were placed. Neurological evaluation showed a minimal conscious state. She presented high fever on the 35th intensive care unit day. Cerebrospinal fluid was sampled and the external ventricular catheter was removed. *Enterococcus faecalis* was isolated from the culture specimen. The patient received targeted treatment with an ampicillin plus ceftriaxone combination, and a follow-up culture confirmed the pathogen’s eradication. Although she was considered cured, she had a prolonged intensive care unit stay and finally died in the ward two months after the completion of treatment. This case highlights the first reported use of this combination in a severe, non-endocarditis, invasive enterococcal infection, while the review discusses treatment options for nosocomial ventriculitis/meningitis.

## 1. Introduction

Ventriculo-meningitis (VM), or nosocomial or post-operative meningitis/ventriculitis, is a severe infection that complicates neurosurgical surgery, external ventricular (or lumbar) drainage, and ventriculoperitoneal shunts [1]. The incidence of this infection varies from 0 to 22%, and it is due to either Gram-negative or Gram-positive bacteria [1,2,3]. Members of the genus *Enterococcus* are resilient to the healthcare environment, intrinsically resistant to many antimicrobial agents, and can cause device-associated infections [4]. *Enterococcus faecalis*, which causes about two-thirds of enterococcal infections, was the fifth most frequent pathogen isolated from bloodstream infections worldwide [5,6]. In contrast to *Staphylococcus* spp., *Enterococcus* spp. strains are less commonly isolated from cerebrospinal fluid cultures of nosocomial VM patients. In a case series from Spain, the authors estimated the occurrence rate of enterococcal meningitis, community-acquired and nosocomial, at 0.3–4% [7]. Clinical or epidemiological criteria cannot be used to distinguish enterococcal VM (EVM) from VM because of the presence of other bacteria [8,9].

The species and presence of resistance mechanisms determine the suggested antimicrobial chemotherapy [10,11]. Most *E. faecalis* isolates are susceptible to aminopenicillins, while most *Enterococcus faecium* isolates are not. Aminopenicillins such as ampicillin and amoxicillin are the preferred antimicrobials for treating susceptible enterococcal infections in non-allergic patients [11]. Apart from aminopenicillins, available active antimicrobial agents are linezolid, imipenem, vancomycin, teicoplanin, and fosfomycin; however, antimicrobial resistant rates vary across countries around the world [12]. *E. faecium* is one of the most common pathogens responsible for nosocomial infections and is multi-resistant to antibacterial action [13]. The recommended treatment is vancomycin or, in the presence of the vancomycin-resistant phenotype, linezolid or daptomycin [11]. Combining ampicillin or vancomycin with an aminoglycoside to enhance bactericidal action is the routine treatment for more invasive infections such as endocarditis and meningitis [4].

Nonetheless, the treating physician must consider that the central nervous system’s barriers hinder antibacterials from entering the cerebrospinal fluid compartment following their systematic administration [14]. Meanwhile, the current Infectious Diseases Society of America guidelines for healthcare-associated ventriculitis and meningitis do not provide suggestions for treating EVM [15]. However, clinicians can draw expertise from invasive enterococcal infections at other sites, such as infective endocarditis, where ampicillin is combined with an aminoglycoside or ceftriaxone [10,16,17]. Ceftriaxone is a standard meningitis therapy, as it is a poor ligand for the transporters (primarily located in the choroid plexuses) that actively carry away from the central nervous system and many other members of the beta-lactam class [18]. Meanwhile, daily ampicillin dosing for VM may be as high as 15 g or even more, particularly in the critical care setting, where its volume of distribution is increased and renal clearance is augmented [14,19]. An ampicillin–ceftriaxone treatment for complicated bacteremia and endocarditis was associated with fewer relapses than ampicillin monotherapy [10,20].

The objective of the present report is to discuss the therapeutic management of nosocomial enterococcal VM considering there are limited data on the subject. Notably, we present a successful bacterial eradication in an *E. faecalis* VM case with a combination of ampicillin and ceftriaxone.

## 2. Description

A 68-year-old woman was admitted to the neurosurgical department for a scheduled resection of a left sphenoid wing meningioma. She presented with a medical history of insulin-dependent diabetes mellitus, hypertension, and coronary artery disease, and had no allergies. She developed right upper and lower arm weakness and was unable to walk. After the surgery, she was transferred to the intensive care unit. The following day, she remained unconscious after sedation withdrawal, and a repeat computerized tomography scan revealed hemispheric hemorrhage and a left middle cerebral artery pseudoaneurysm. The latter was stented and an external ventricular drainage catheter was placed. During the following weeks, neurological evaluation showed a minimally conscious state.

On the 35th day in the intensive care unit, she developed a high fever after the removal of the external ventricular catheter the day before. Cerebrospinal fluid (CSF) specimens were sent for culture and routine analysis (Table 1).

The following day, a Gram-positive microorganism was isolated from the CSF culture. On day 22 of drainage, the external ventricular drainage catheter was suspected to be the primary source of the VM, but the two craniotomies could not be ruled out as contributing causes of the infection. The removal of the catheter was considered the initial therapeutic step. Regarding antimicrobial treatment, linezolid (Demo Pharmaceuticals, Athens, Greece) was started, pending further laboratory input. *E. faecalis* was identified by matrix-assisted laser desorption ionization performed by the MALDI Biotyper^®^ Sirius System (Bruker, Bremen, Germany). Antimicrobial susceptibility testing performed by the Vitek^®^2 automated system (bioMerieux, Marcy-l;Etoile, France) showed susceptibility to ampicillin and high-level gentamycin resistance. Ampicillin 16 g daily (Cooper Pharmaceuticals, Athens, Greece) by continuous infusion plus ceftriaxone 2 g (Anfarm Pharmaceuticals, Athens, Greece) twice daily were substituted for linezolid. Meanwhile, creatinine clearance, measured after 24 h urine collection, was augmented at 190 mL/min. The patient’s fever subsided in 4 days, following treatment. A follow-up CSF culture and routine fluid examination on treatment day 13 confirmed bacterial eradication (Table 1). The treatment lasted for 14 days. Following the pathogen clearance, the patient remained afebrile, and her neurological status failed to improve. Ventilator weaning was prolonged, lasting five weeks. She was then transferred to a neurosurgical ward, where she died three weeks later, and two months after the completion of treatment, without showing any signs of infection.

## 3. Discussion

The literature on nosocomial EVM is summarized in Table S1 [7,8,9,21,22,23,24,25,26,27,28,29,30,31,32,33,34,35,36,37,38,39,40,41,42,43,44,45,46]. Previous studies were usually small case series and case reports, while there were only four series with at least ten cases each. Of 138 reported cases, a species was identified in 128. *E. faecalis* was most commonly identified (59.4%), followed by *E. faecium* (26.1%). Almost all *E. faecalis* isolates retained susceptibility to ampicillin, whereas vancomycin-resistant *Enterococcus* (VRE) was isolated in 23.2% of patients. Finally, there were 55 (39.9%) pediatric patients. Regarding treatment options, there are antimicrobials considered more efficacious that present favorable pharmacokinetic profiles, i.e., adequate penetration into the CSF after intravenous delivery. However, when one of the above agents cannot be administered, there are less optimal alternatives (Table 2).

Among the most commonly used agents, linezolid is a suitable empirical treatment for nosocomial Gram-positive VM as it retains activity against VRE and methicillin-resistant *Staphylococcus* and sufficiently enters the CSF following its intravenous (IV) administration [47,48,49,50]. Moreover, limited efficacy data further support its use for treating EVM (Table 2).

The gold standard for enterococcal infections is ampicillin whenever susceptible strains are involved [10,11]. Concerning the management of EVM, it was often combined with aminoglycosides and only once with cefotaxime (Table 2 and Table S1). Regarding dosing, a high IV ampicillin dose of 200 mg/kg/day or an adult daily dose of approximately 15 g should suffice [7,14]. The presence of high-level gentamycin resistance in our case made us consider using ceftriaxone as the companion drug. This is, as far as we are aware, the first reported co-administration of ampicillin and ceftriaxone for treating EVM.

Vancomycin, in combination with a beta-lactam class antipseudomonal drug, is recommended for empirically treating VM [15]. However, the current guidelines recommend the use of vancomycin in the context of staphylococcal infections, particularly if they are methicillin-resistant. The drug dose should be adjusted to maintain trough levels of 15–20 mcg/mL [15]. Data on vancomycin use in EVM are limited to case series and case reports (Table S1). Although IV vancomycin demonstrated unpredictable CSF penetration, most patients were cured [51]. Teicoplanin showed similar efficacy to vancomycin for managing methicillin-resistant *Staphylococcus aureus* infection [52]. However, its role in treating EVM is not defined.

Regarding less common options, daptomycin can be used to manage bloodstream VRE infections; concerning EVM, daptomycin poorly penetrates the CSF and is rarely used [1,37,53,54,55,56]. The efficacy of tigecycline has been questioned, as it demonstrated increased mortality compared with other antimicrobials; moreover, it poorly enters the CSF following IV delivery [57,58]. Thus, it can be combined with other active drugs as salvage therapy [57]. It has been used for treating VM, due to extensively drug resistant Gram-negative pathogens, via both IV and IT/IVT routes [1,59,60]. Currently, there are no available data for tigecycline treatment for EVM. In vitro data showed that chloramphenicol often retains activity against *E. faecium* regardless of vancomycin resistance, although enterococcal resistance rates to the drug vary widely between countries and have increased over time [12,61]. Meanwhile, this drug demonstrated favorable penetration into the CSF (approximately 50%) and had comparable efficacy to beta-lactams in meningitis treatment [1,62]. However, adding IT/IVT to IV chloramphenicol may improve efficacy (Table 2), while toxicities, particularly hematological, are a major concern [11]. Fosfomycin adequately penetrates the CSF, and has been used to treat mostly non-enterococcal central nervous system infections with a companion antimicrobial in 87% of patients [63,64]. This combination regimen achieved a cure rate as high as 90% and a CSF sterilization rate of 97% [63]. It is active against enterococci, including VRE, and could be a valuable therapeutic option for EVM, given its favorable CSF entry profile. Quinupristin/dalfopristin is inactive against *E. faecalis*, but retains activity against most *E. faecium* strains [11]. Regrettably, it was associated with higher mortality than linezolid and its indication for treating VRE bloodstream infections was withdrawn. It presents severe adverse effects and should be used, if available, as a complementary drug to a combination regimen [11]. Nonetheless, it minimally enters the CSF and should seldom be considered a suitable treatment option [65].

There are novel antimicrobials that include enterococci in their antimicrobial spectrum that might be used in the management of EVM [66]. Eravacycline, a tetracycline related to tigecycline, does not sufficiently enter the CSF. Similarly, the novel glycopeptides, either dalbavancin or telavancin, or oritavancin, are poorly transferred to the CSF after IV administration. Finally, tedizolid, although closely related to linezolid, penetrated the CSF less than linezolid, lacking the latter’s excellent pharmacokinetic profile for treating CNS infections [66].

Finally, adjunct IT/IVT therapy is directly delivered to the CSF compartment, bypassing the blood–brain and blood–CSF barriers. Regarding EVM, there are limited literature data on adjunct IT/IVT treatment with vancomycin or aminoglycoside, or, more recently, daptomycin (Table S1).

## 4. Summary

In line with previous reports on EVM treatment, which described the use of ampicillin either as the sole antimicrobial or complemented with an aminoglycoside, we found that the combination of ampicillin and ceftriaxone eradicated the pathogen in the CSF. The literature on EVM mostly includes small case series and case reports on various antimicrobial options for treating enterococcal strains. Enterococci are endogenously resistant to a plethora of antimicrobial agents, while available in vitro active drugs should penetrate the blood–brain and blood–CSF barriers to reach the infection site. In addition to *E. faecalis*, which usually remains susceptible to ampicillin, many reports discuss the more resistant *E. faecium* species, which can be successfully treated with IV linezolid. Other less commonly used drugs, particularly for treating VRE infections, are daptomycin, chloramphenicol, and quinupristin/dalfopristin. Daptomycin should preferably be delivered by both IV and IT/IVT routes to achieve sufficient levels in the CSF compartment. Regarding the prognosis of EVM, mortality varies between reports; however, there is uncertainty about the outcome because of the very low quality of evidence (Table 2).

## 5. Conclusions

The present case highlights the treatment of enterococcal ventriculitis/meningitis, an uncommon infection, with a combination of ampicillin and ceftriaxone. Despite the infection eradication, the patient died two months later; however, she did not present any evidence of infection at the time of her death. Further studies are needed to better define the generalizability and practical aspects of this antimicrobial scheme for the management of central nervous system infections.

## Figures and Tables

**Table 1:** CSF analysis and culture.

	Before Treatment	13th Treatment Day
WBC, *n*/μL	2000	17
Glucose, mg/dL	3	108
Protein, mg/dL	252	264
Culture	*E. faecalis*	no isolate

Abbreviations: WBC, white blood cells.

**Table 2:** Treatment options for nosocomial enterococcal ventriculitis/meningitis according to previous reports.

Primary Antimicrobial Agent	Most Common Companion Antimicrobial	Target Species/Susceptibility to the Agent	CSF Penetration	Frequency of Use *	Dose	Duration	Mortality #
***Most commonly used primary agents***							
Ampicillin	AG	*Enterococcus faecalis*/yes	Relatively low; the daily dose can be increased to improve delivery	37%	Adult, 15 g (or more); child, 200 mg/kg/day	Median (IQR) 21 (15–24) days	7.7%
Linezolid	Occasionally rifampicin, IT/IVT daptomycin	*Enterococcus faecium*/yes, including VRE	Excellent	12.3%	Adult, 600 mg ×2	14–28 days	0%
Vancomycin	Variable: carbapenem, AG, cefalosporin	*E. faecalis*/yes *E. faecium*/yes, except VRE	Unpredictable	26.1%	Loading dose, adult, 15–30 mg/kg; followed by adult, 30–60 mg/kg/day; child, 60 mg/kg/day §	NS	10%
***Less common primary agents***							
Daptomycin	Gentamycin, linezolid, IT/IVT daptomycin	*E. faecium*/VRE included	Poor	2.9%	6–12 mg/kg	20–30 days	0%
Quinupristin/dalfopristin	IT/IVT quinupristin/dalfopristin, linezolid	*E. faecium*/VRE included	Poor	2.9%	7.5 mg/kg thrice daily	Variable	50%
Chloramphenicol	IT/IVT chloramphenicol	*E. faecium*/VRE often included	Excellent	2.2%	Adult, 3 g daily; child, 100 mg/kg/day	12–27 days	0%

* The denominator is 138, the total number of reported cases. # Outcome evaluation is limited as the number of cases is small. § The post loading dose can be given by a continuous infusion; when it is administered by intermittent infusion, adjust dosing to maintain vancomycin’s trough concentrations of 15–20 μg/mL. Abbreviations: AG, aminoglycoside; IT, intrathecal; IVT, intraventricular; NS, not specified; VRE, vancomycin-resistant *Enterococcus*.

## Data Availability

The original contributions presented in this study are included in the article/Supplementary Materials. Further inquiries can be directed to the corresponding author.

## Supplementary Materials

The following supporting information can be downloaded at: https://www.mdpi.com/article/10.3390/pathogens14010003/s1, Table S1: Enterococcal ventriculitis/meningitis reports in the literature.
