# Supplementary material for: Treatment Options for Nosocomial Ventriculitis/Meningitis: A Case Report and Review of the Literature
Source: Pathogens. 2024 Dec 26;14(1):3. doi: 10.3390/pathogens14010003 (PMC11768174; doi:10.3390/pathogens14010003)
Supplement: Supplementary file 1 [file pathogens-14-00003-s001.zip › pathogens-3375499-supplementary.pdf]

SUPPLEMENTARY MATERIAL

Table S1. Enterococcal ventriculitis/meningitis* reports in the literature.

Author	Country	Year	Cases, number	Adult / child	Post-neurosurgical, number	EVD/VPS on diagnosis §	Enterococcus species	Resistance traits$	IV treatment	IT treatment	Survival status†
Pintado	Spain	1976-2000	39	31/8	33	27 total devices	*E. faecalis* (90%) #	10% AMP-resistant	First line drug: AMP 64.1%, VAN 23.1% Combo drug: aminoglycoside 46.2%	20.5% VAN or aminoglycoside	Mortality 26%
Rodriguez Guardado	Spain	1994-2003	20	20/-	20	60% /15%	*E. faecalis* (90%)	None for the *E. faecalis* isolates; no VRE isolate was detected	13, pts VAN, 12 with another antimicrobial ! 6, AMP (1, plus cefotaxime) 1, imipenem	25%, VAN	Mortality 20%
Losonsky	USA	1994	1	-/yes	1	no/no	*E. faecium*	VRE	Rifampicin+ AMP+Clindamycin	Teicoplanin	Survived
Stevenson	USA	1994	4	4/-	1	a patient had lumbar drain	NS	no	3, AMP+GENT 1, VAN+GENT	1, GENT	Mortality 0%
Nachman	USA	1995	1	-/yes	1	-/yes	*E. faecium*	VRE	quinupristin/ dalfopristin	quinupristin/dalfopristin	Survived
Tan	UK	1998	1	yes/-	1	yes/-	*E. faecium*	VRE	quinupristin/ dalfopristin	quinupristin/dalfopristin	Died
Tush	USA	1998	1	yes/-	1	yes/-	*E. faecium*	VRE	quinupristin/ dalfopristin	quinupristin/dalfopristin	Died
Perez Mato	USA	1999	1	-/yes	1	-/yes	*E. faecium*	VRE	chloramphenicol	-	Survived
Shaikh	USA	2001	1	yes/-	1	yes/-	*E. faecium*	VRE	LINE	-	Survived
Steinmetz	USA	2001	1	yes/-	1	yes/-	*E. faecium*	VRE	LINE	-	Survived
Graham	USA	2002	1	-/yes	1	-/yes	*E. faecium*	VRE	LINE	-	Survived
Schnapp	Israel	2002-23	10	-/10	5	-/5	*E. faecalis* (90%) *E. faecium* (10%)	no	First line drug: AMP 70%, VAN 20% Combo drug: aminoglycoside 20%	-	Mortality 0%
Kanchanapoom	USA	2003	1	yes/-	1	Ommaya reservoir	*E. faecium*	VRE	Quinupristin /dalfopristin plus linezolid	Quinupristin/dalfopristin	Survived

Scapellato	Argentina	2005	1	yes/-	1	no/no	*E. faecium*	VRE	Chloramphenicol	Chloramphenicol	Survived
da Silva	Brazil	2007	1	-/yes	1	yes/-	*E. faecium*	VRE	LINE	-	Survived
Elvy	UK	2007	1	yes/-	1	yes/no	*E. faecalis*	no	DAPTO	DAPTO	Survived
Maranich	USA	2008	1	-/yes	1	-/yes	*E. faecium*	VRE	LINE	-	Survived
Hartmann	Germany	2010	1	-/yes	1	Rickham reservoir	*E. faecium*	VRE	chloramphenicol	chloramphenicol	Survived
Le	USA	2010	3	3/-	3	-/33%	*E. faecium*	VRE	DAPTO	-	Mortality 0%
Cay	Turkey	2011-2021	24	-/24	22	14 pts, hydrocephalus shunt	*E. faecalis* (44%)<br>*E. faecium* (44%)<br>*E. gallinarum* (12%)	16.7% ampicillin resistant, 20.8% VRE	VAN or LINE, 87.5% combined with a Gram-negative active agent¶	14 pts;<br>10, VAN+AMI<br>1, VAN<br>1, VAN+GENT<br>2, LINE	Mortality 8,3%
Mueller	USA	2012	1	yes/-	1	yes/-	*E. faecium*	VRE	LINE	DAPTO	Survived
Knoll	USA	2013	4	4/-	3	NS	*E. faecium*	VRE	1, LINE<br>2, LINE plus rifampin<br>1, Quinupristin-dalfopristin plus DAPTO	-	Mortality 25%
Khanum	Pakistan	2013-2016	6	3/3	6	33%/33%	NS	All AMP-resistant, 33% VRE	4 pts, VAN<br>1, LINE<br>1, LINE plus rifampin	no	Mortality 0%
Qiu	China	2015	1	yes/-	1	no/no	*E. faecium*	VRE	LINE	-	Survived
Youcef Khan	Qatar	2021	8	5/3	6	100	*E. faecalis* (87.5%)	no	AMP, 50% solely & 50% combined with VAN or meropenem	-	Mortality 0%
Cearns	UK	2022	1	yes/-	1	yes/-	*E. faecium*	VRE	LINE	no	Survived
Dhariwal	UK	2022	1	yes/-	1	yes/-	*E. faecium*	VRE	LINE	DAPTO	Survived
Rei	USA	2023	1	yes/-	1	-/yes	*E. faecium*	VRE	LINE	DAPTO	Survived
Inada	Japan	2024	1	yes/-	1	no/no	*E. faecalis*	no	AMP + GENT	no	Survived

*Nosocomial or postoperative or post-neurosurgical ventriculitis/meningitis is the focus of this report. All single case reports are nosocomial.
§Regarding nosocomial cases only
$Regarding AMP and VANCO
†Follow-up varied between studies
#11 mixed infections, 6 with Gram-negative pathogens
! Imipenem, meropenem, amikacin, cefotaxime, ceftazidime, cefipime
¶Meropenem, ceftriaxone, ciproxin, cefipime, ceftazidime, trimethoprim/sulfamethoxazole
Abbreviations: AMP, ampicillin; DAPTO, dapto; EVD, external ventricular drainage; GENT, gentamycin; IT, intrathecal; IV, intravenous; LINE, linezolid; NS, not specified; pt, patient; VAN, vancomycin; VPS, ventriculoperitoneal shunt; VRE, vancomycin-resistant *Enterococcus*
